# Stunting and associated factors among children aged 6–59 months from productive safety net program beneficiary and non-beneficiary households in Meta District, East Hararghe zone, Eastern Ethiopia: a comparative cross-sectional study

**DOI:** 10.1186/s41043-022-00291-0

**Published:** 2022-04-05

**Authors:** Aklilu Tesfaye, Gudina Egata

**Affiliations:** 1 Chelenko District Health Office, Chelenko Town, East Haraghe Zone, Ethiopia; 2 Department of Nutrition and Dietetics, School of Public Health, College of Health Sciences, Addis Ababa University, Addis Ababa, Ethiopia

**Keywords:** Children 6 to 59 months, Ethiopia, PSNP, Stunting

## Abstract

**Background:**

Undernutrition is one of the major public health problems affecting children in developing settings. Despite impressive interventions like productive safety net program (PSNP), there is limited information on the association between stunting and PSNP implementation in Ethiopia.

**Methods:**

Community-based comparative cross-sectional study design was used among systematically selected 1555 children and their mothers/caregivers from households enrolled in PSNP and not, respectively, in Meta District east Ethiopia from 5th–20th of March 2017. Data were collected using pretested structured questionnaire. Measuring board was used to measure length/height of children. Length/height for age *Z*-score was generated using World Health Organization (WHO) Anthro version 3.2.2. Descriptive statistics was used to describe all relevant variables. Bivariable and multivariable logistic regression analyses were used to identify predictors of stunting. Odds ratio along with 95% confidence intervals were estimated to measure the strength of association. The statistical significance was declared at *p* value less than 0.05.

**Results:**

The prevalence of stunting was 47.7%, 95% CI (44.1%, 51.5%) and 33.5%, 95% CI (29.9%, 36.9%) among children from households enrolled in PSNP and non-PSNP ones, respectively. Lack of maternal education [AOR = 3.39; 95% CI (1.12, 5.11)], women’s empowerment [AOR = 3.48; 95% CI (2.36, 5.12)] and fourth antenatal care visit [AOR = 4.2, 95% CI (2.5, 6.8)], practicing hand washing [AOR = 0.46; 95% CI (0.28, 0.76)], living in mid-land [AOR = 1.94, 95% CI (1.12, 3.35)] and low-land[AOR = 0.27: 95% CI (0.16, 0.45)] agro-ecological zones, PSNP membership [AOR = 1.82, 95% CI (1.14, 2.89)], childhood illness [AOR = 8.41; 95% CI (4.58, 12.76)], non-exclusive breastfeeding [AOR = 3.6; 95% CI (2.30, 4.80)], inadequate minimum dietary diversity [AOR = 4.7; 95% CI (3.0, 7.40)], child’s sex [AOR = 1.73, 95% CI (1.18, 2.53)] and age (24–59 months) [AOR = 3.2; 95% CI (1.6, 6.3)] were independent predictors of stunting.

**Conclusions:**

The prevalence of stunting was high among children from households enrolled in PSNP. Stunting was significantly associated with maternal- and child-related factors. Therefore, women empowerment on household’s issues and improving infant and young child feeding practices could reduce the prevalence of stunting and its adverse consequences.

## Background

Undernutrition is known to be one of important causes of childhood illness, disease, and disability among children of low-income countries. Undernourished children are victims of various deficiency states such as night blindness, anemia, iodine deficiency disorder, mental retardation and risk of dying compared with well-nourished children. Globally, 22.5% of under-five children are stunted. South Asia and sub-Saharan Africa (SSA) including Ethiopia have the greatest lion share of the burden of undernutrition [1, 2].

Stunting was one of the major nutritional problems of public health importance in Ethiopia, where 38%, 24%, and 10% of children aged 0–59 months were stunted, underweight and wasted, respectively, though there is marked reginal variation within the country [3, 4].

The causes of undernutrition including stunting are multifaceted in SSA including basic, underlying and proximal factors. Different studies indicated that undernutrition is attributed to maternal illiteracy, large family size, maternal age., being male child, child birth order, amount of water (< 40 l) for use, lesser child age, lack of extra food during pregnancy/lactation, and low dietary diversity score (DDS below four food groups) due to household food insecurity among others in developing countries like Ethiopia [3–8].

In cognizant of this, the government of Ethiopia has launched productive safety net program (PSNP), one of the social protection programs implemented in SSA, since 2005 being implemented in different phases as a strategy to reduce the burden of undernutrition by transferring cash and food to poor food-insecure rural households through public works of able bodied labors and direct transfers for none adult able body labor in the households [9–11], thus improving availability and access to adequate and nutritious food. The program has public work, direct support and institutional support. Some of the public work activities include water point development, road maintenance, agro-forestry, irrigation canal, schools and health post maintenance & construction [12]. The national nutrition program (NNP II) was planned to be implemented in Ethiopia from 2016 to 2020, including health all sectors should increase their efforts to enhance good nutritional practices through different interventions in reducing and preventing malnutrition [13].

However, there is a paucity of evidence with regard to stunting and its predictors among children aged 6–59 months who live in productive safety net program beneficiary’s households and non-beneficiary's  households in Ethiopia particularly in the study setting. Therefore, this study was aimed at assessing the prevalence of stunting and its predictors among children aged 6–59 months who belong to PSNP beneficiary and non-PSNP beneficiary households.

## Methods

### Study setting, period, and design

Community-based comparative cross-sectional study design was used in Meta District, East Harerge Zone, Eastern Ethiopia, from 5th to 20th of March 2017. The district has a total population of 222,016 (28,620 urban and 193,396 rural population) [14]. Chelenko is the main town of district which is 435 km far away from Addis Ababa, the capital city of Ethiopia. The district has 39 rural and 3 urban kebeles (smallest administrative unit in Ethiopia). The rural kebeles of the district were divided into three agro-ecological zones (12 high-lands, 15 mid-lands and 12 low-lands kebeles). The main ethnic groups in the district are Oromo and Amhara. The livelihood of the rural population was farming. In the district, there are 1 hospital, 7 health centers, 39 rural health posts, and 11 different private health facilities rendering health services. According to the 2017 report of the District disaster prevention and preparedness office (DPPO) [15], the district has 23 foods insecure rural kebeles that have been covered with PSNP and 16 food secure households. The total number of PSNP beneficiary households was 18,948, 17.5% of the district’s population.

### Sample size determination, study participants, and sampling procedure

**Fig. 1:**
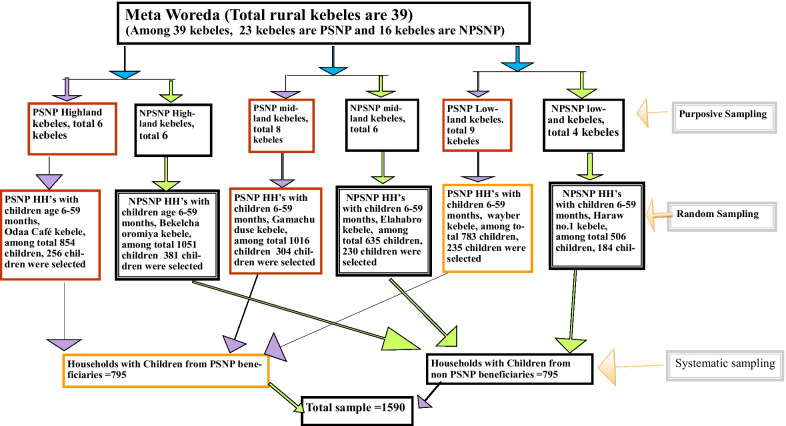
Schematic sampling procedure to select the study participants from both PSNP and NPSNP beneficiaries in Meta District, March 2017

The sample size was estimated using double population proportion formula for both PSNP beneficiary and non-beneficiary households with the following assumptions: two sided 95% confidence level, 80% power, proportion of moderate stunting among children from PSNP beneficiary households to be 47.0% and proportion of moderate stunting among PSNP non-beneficiary households to be 41.8% based on evidence from nation-wide community-based survey [3], desired precision of 5%, and 10% for non-response yielding 1590 children aged 6–59 months and their mothers/care givers (Mother/caregiver—pair) from Productive Safety Net beneficiary (795) and non-Productive Safety Net (795) beneficiary households in the district. The sample size was then allocated for each selected kebeles in each arm proportional to their population size. Initially, the rural kebeles were stratified in to high-land, mid-land and low-land agro-climatic zones based on the ecology of the study area. All children aged 6 to 59 months and their mothers/care takers (mother/care giver–child pair) in selected PSNP beneficiary and non-PSNP beneficiary households who lived in the district for at least six months were considered as the study population. Overall, there are 23 rural kebeles benefiting from PSNP and 16 rural kebeles not part of the program. For this survey, a total of 7 kebeles (3 kebeles from non-PSNP beneficiaries and 4 kebeles from PSNP beneficiaries) were selected using a stratified random sampling method using climatic zone as a stratum to take part in the study. Children and their mothers/care givers from households in each respective kebele were selected using systematic sampling for both PNSP and NPNP households after determining the sampling interval which was 3. The sampling interval was obtained by dividing the total number of households in both population segments by their respective sample size. After deciding the random start, samples were identified until the required samples were reached (Fig. 1).

### Data collection tool and measurements

The questionnaire was initially prepared in English by reviewing relevant literature related to the study objectives and translated to “afaan Oromo,” the regional language of the study setting, for better understanding and translated back to English by two different experts who were fluent in both languages in order to ensure the consistency of the questionnaire. The questionnaire consisted of seven sections namely mother’s socio-demographic characteristics, household’s properties (assets) related questions, household’s PSNP membership status, mother’s reproductive health services and related characteristics, child feeding practices, child’s history of illness two weeks prior to the survey, and child’s height measurement and reported age. Fourteen data collectors and two supervisors were involved in the process of data collection after getting the required training with regard to interview and anthropometric measurements. Length/height was measured following the standard procedures to the nearest 0.1 cm using wooden measuring board produced under the guidance of United Nations Children's Fund (UNICEF). Recumbent length was measured for children younger than 24 months while height was measured for those children 24 months and older in a standing position for all who can stand well by maintaining five contact points [16].

However, some adjustment was made for children whose mothers were not able to gauge the exact age of their children to decide between recumbent and standing measurement. In some cases, local calendar was used to estimate the age of the child when possible or estimation of age was made from length of the child in centimeter as per WHO recommendation. The child is considered to be younger than 24 months if his or her length is less than 87 cm and 24 months and above if his or her length is greater or equal to 87 cm using tape measure [17].

The outcome variable in this study was stunting which stands for children having length/height for age *Z*-score (HAZ) of less than minus 2 standard deviation (SD) using WHO new child growth. Stunting was coded as “1” for children with HAZ of less than − 2SD and “0” otherwise for further analysis [18].

Dietary diversity among the study population was measured following WHO’s infant and young child feeding (IYCF) recommendation which states that percentage of children 6–23 months of age who consumed foods and beverages from at least four out of seven defined food groups according to the former definition during the previous day which has been used for this study. Such children are said to have adequate dietary diversity compared with their counterparts who consumed foods from less than four food groups [19].

### Data quality management

Training was given for the data collectors and supervisors for two days on the objectives of the study, interview technique, and anthropometric measurements. The standardization procedure was followed to ensure reliability and validity of anthropometric measurements by computing relative technical error of measurement (TEM) using Emergency Nutrition Assessment Standardized Monitoring and Assessment of Relief and Transitions (ENA SMART) software to compare measurements done by each data collector with selected criterion anthropometrist before deploying the data collectors to the field to minimize both random and systematic errors attributed to inaccurate anthropometric measurement. Accordingly, the relative TEM for interobserver (validity) and intraobserver (reliability) for length/height measurement was 1.5% and 2.0%, respectively [20]. The questionnaire was pretested one week before the actual task of data collection on 5% of the estimated sample size for the study in kebeles not included in the study. The whole process of data collection was supervised by supervisors in the field on daily basis for completeness of each questionnaire. Data were double entered by two data clerks and consistency was crosschecked. Multivariable analysis was done to control for all possible confounders that might mask the true association between independent and outcome variable.

### Statistical analysis

Data were manually checked for completeness, edited, double entered onto Epi-Data version 3.1, cleaned, and exported to Statistical Package for the Social Sciences(SPSS) version 20 computer software for further analysis. Moreover, anthropometric indices were also generated using WHO Anthro software version 3.2.2 and coded based on WHO’s cut-off points to serve as outcome variable. Descriptive statistics such as numerical summary measures and frequency distribution was used to describe each independent variable relative to the outcome variable. The independent variables were coded based on previous related studies and distribution of responses in the data. Bivariable logistic regression analyses were done to see the association between dependent variable and each independent variable. All covariates with *p* value < 0.25 during bivariable analyses were considered for multivariable analysis to control for all possible confounders and to identify predictors of stunting. Principal component analysis (PCA) was done to generate the wealth index of the households. The multicollinearity effect between independent variables was checked by looking at values of the standard error and correlation coefficient. Variables with standard error of > 2 and correlation coefficient greater than 50% were dropped from final binary logistic regression model. The fitness of the model was tested by Hosmer–Lemeshow goodness of fit test with a *p* value equals 0.631 indicating the fitness of model to the data. Odd ratios along with 95% confidence interval were estimated to measure the strength of the association. Level of statistical significance was declared at *p* value less than 0.05.

## Results

### Socio-demographic characteristic of the study participants

A total of 1555 (97.8%) of respondents were participated in the study making a response rate of 97.8% of which 782 (98.4%) participated from PSNP while 773 (97.2%) participated from NPSNP. Among 782 PSNP respondents, 153 (19.6%), 403 (51.5%) and 226 (28.9%) were participated from high-land, mid-land and low-land respectively. Among 773 NPSNP respondents, 390 (50.5%), 224 (29%) and 159 (20.6%) were participated from high-land, mid-land and low-land, respectively. The mean (± SD) age of mothers/caregivers from the high-land was (29.0 ± 6.34) years, mid-land (30 ± 6.68) years and low-land (30 ± 6.678) years respectively. Most of the respondents 1504 (96.7%) was Oromo by their ethnicity while 51 (3.3%) was Muslim by their religion. Nearly 94% of the mothers/care takers were married. About 11.6% of the mothers and 25.5% of their husbands have formal education. The majority (89.8%)) of the mothers were house wives by their occupation while 95.8% of their husbands were farmers. The majority (27.2%) of the mothers/caregivers belong to the poorest households according to their wealth index status. Nearly equal proportion of mothers/caregivers were from the three agro-ecological areas though most of the mothers from PNSP programs belong to the poorest segment (40.2 %,) compared to those from NPSP (14.1%) (Table1).

**Table 1:** Socio-demographic characteristics parents with children aged 6–59 months, Meta District east Ethiopia 2017

Variables (*n* = 1555)	PSNP HH's	NPSNP HH's	*p* value
	n (%)	N (%)	
Mother education status			
Have no formal education	685 (87.6)	571 (73.9)	0.001
Read and write only	27 (3.5)	90 (11.6)	
Primary school	51 (6.5)	53 (6.9)	
Secondary school	19 (2.4)	51 (6.6)	
Diploma/degree	0 (0)	8 (1)	
Father education status			
Have no formal education	505 (64.6)	442 (57.2)	0.0001
Read and write only	112 (14.3)	100 (12.9)	
Primary school	132 (16.9)	113 (14.6)	
Secondary school	25 (3.2)	83 (10.7)	
Preparatory	8 (1)	21 (2.7)	
Diploma/degree	0 (0)	14 (1.8)	
Maternal occupation			
Home lady	676 (86.4)	721 (93.3)	0.0001
Farmer	12 (1.5)	11 (1.4)	
Teacher	0 (0)	12 (1.6)	
Civil servant	0 (0)	4 (0.5)	
Merchant	94 (12)	25 (3.2)	
Paternal occupation			
Farmer	782 (100)	708 (91.6)	0.0001
Civil servant	0 (0)	16 (2.1)	
Teacher	0 (0)	17 (2.2)	
Merchant	0 (0)	12 (1.6)	
No job (jobless)	0 (0)	20 (2.6)	
Ethnic group			
Oromo	772 (98.7%)	732 (94.7%)	0.0001
Amhara	10 (1.3%)	41 (5.3%)	
Marital status			
Married	723 (92.5%)	724 (93.7%)	0.0001
Divorced	4 (.5%)	13 (1.7%)	
Widowed	43 (5.5%)	11 (1.4%)	
Separate	12 (1.5%)	25 (3.2%)	
Mother’s age			
18–44 years	742 (95.3%)	762 (98.6%)	0.0001
45 and above years	40 (5.1%)	11 (1.4%)	
Family size			
0–4 family	127 (16.2)	399 (51.6)	0.0001
5–8 family	528 (67.5)	321 (41.5)	
9 and more	127 (16.2)	53 (6.9)	
Agro–ecologic zone			
High-land	153 (19.6%)	390 (50.5%)	0.0001
Low-land	403 (51.5%)	224 (29%)	
Mid-land	226 (28.9%)	159 (20.6%)	
Wealth index			
Poorest	314 (40.2)	109 (14.1)	0.0001
Poor	128 (16.4)	92 (11.9)	
Medium	174 (22.3)	158 (20.4)	
Rich	138 (17.6)	292 (37.8)	
Richest	28 (3.6)	122 (15.8)	

### Maternal reproductive health-related characteristics and Child feeding practices

About half (55.2%) of women attended antenatal care service up to 4 visits (ANC IV) at health facilities; (49.3% in PSNP and 61.4% in NPSNP). Almost two-third (63.7%) of the women were utilizing family planning services, (56.3% in PSNP and 71.3% in NPSP households). The most commonly used birth control methods were Depo-Provera (40.8%) followed by implant (17.8%). About 42.6% of the women were married before 18 years. The mean (± SD) age at first marriage was 17.65 (± 2.279) years. About 59.7% of the married women gave birth to the first baby prior to celebrating their 20 years’ birthday. The mean (± SD) age at the first delivery for the mothers was 19.16 (± 2.617) years. Almost all mothers (98.1%) breastfed their children of which only 44.2 % of the mothers-exclusively breastfed their children (47.4% in PSNP and 33.7% in NPSNP households). About 21.4% of mothers started first complementary food at 6 months. The first complementary food given to the children was porridge (55.6%) and animal milk and Porridge (19.4%) respectively. Overall, nearly 44% of children consumed less than four food groups. Forty nine percent of children from PSNP households and nearly 39% from the NPSNP households consumed less than four food groups (Table 2).

**Table 2:** Selected maternal and child’s characteristics among the study participants from PNSP and NPNSP households, Meta District, March 2017

Variables (*n* = 1555)	Member of PSNP		Not member of PSNP		Total		*p* value
	Number	%	Number	%	Number	%	
*Did you have antenatal care follow-up?*							
Yes	422	54	242	31.3	664	42.7	0.0001
No	360	46	531	68.7	891	57.3	
*Did you use birth control?*							
Yes	440	56.3	551	71.3	991	63.7	0.0001
No	342	43.7	222	28.7	564	36.3	
*Method of birth control used*							
IUCD	3	0.4	2	0.3	5	0.3	0.0001
Implant	104	13.3	173	22.4	277	17.8	
Depo-Provera	256	32.7	378	48.9	634	40.8	
Pills	74	9.5	0	0	74	4.8	
*Age of marriage*							
< 18 years	439	56.1	224	29	663	42.6	0.0001
≥ 18 years	343	43.9	549	71	892	57.4	
*Age of 1st delivery*							
< 20 years	497	63.6	431	55.8	928	59.7	0.001
≥ 20 years	285	36.4	342	44.2	627	40.3	
*Place of birth*							
Health facility	503	64.3	537	69.5	1040	66.9	0.018
Home delivery	279	35.7	236	30.5	515	33.1	
*Woman’s participation in decision-making*							
Yes	296	37.9	350	45.3	646	41.5	0.340
No	486	62.1	423	54.7	909	58.5	
*Hand washing practice*							
Yes	171	21.9	122	15.8	293	18.8	0.0001
No	611	78.1	651	84.2	1262	81.2	
*Did a mother breastfeed her children?*							
Yes	771	98.6	752	97.3	1523	97.9	0.0001
No	11	1.4	21	84.2	32	2.1	
*Exclusive breastfeeding*							
Yes	399	51	472	61.1	871	56	0.0001
No	383	49	301	38.9	684	44	
*Age of discontinuation of breastfeeding*							
< 6 months	27	3.5	33	4.3	60	3.9	0.0001
6–11 month	4	0.5	0	0	4	0.3	
12–23 month	751	96	740	95.7	1491	95.9	
*Age of introducing complimentary feeding*							
< 6 months	42	5.4	62	8	104	6.7	0.0001
At 6 Month	426	54.5	548	70.9	974	62.6	
≥ 7 months	314	40.2	163	21.1	477	30.7	
*Minimum dietary diversity*							
< 4 food groups	384	49.1	299	38.7	683	43.9	0.0001
≥ 4 food groups	398	50.9	474	61.3	872	56.1	

### Prevalence of stunting among children aged 6–59 months

**Fig. 2:**
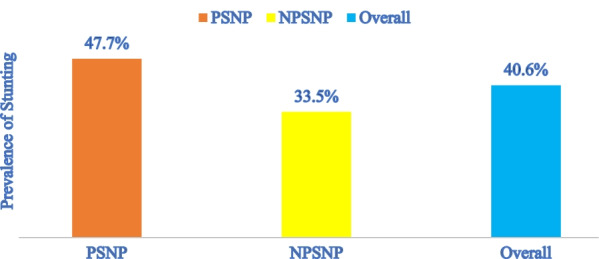
Prevalence of stunting among children aged 6–59 months in PSNP and NPSNP household Meta District, March 2017

The overall prevalence of stunting was 40.6%; 95% CI (38.3%, 43.3%) among children aged 6–59 months in the study setting. The prevalence of stunting was 47.7%; 95% CI (44.1%, 51.5%) among children aged 6–59 months from PSNP beneficiary households and 35.5%; 95% CI (29.9%, 36.9%) among those children from non-PSNP beneficiary households (Fig. 2).

**Fig. 3:**
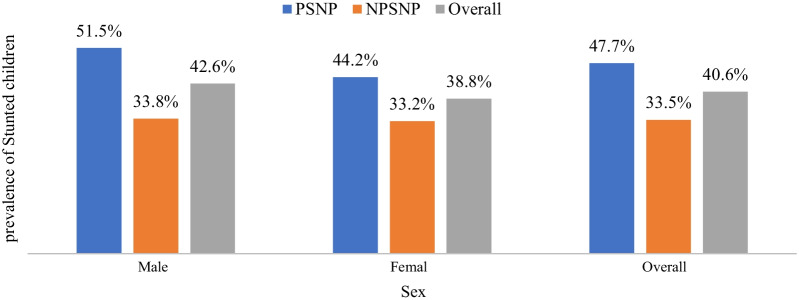
Prevalence of stunting among children disaggregated by sex across PSNP and NPSNP beneficiary households, Meta District, March 2017

This study also showed that male children were more stunted than female children. The prevalence of stunting among male children was 51.5% for PSNP beneficiary households and 33.8% for NPSNP households while the prevalence of stunting among female children was 44.2% for PSNP beneficiary households and 33.2% for NPSNP households. The overall prevalence showed that 42.6% of  male children and 38.8% of female children were stunted (Fig. 3).

**Fig. 4:**
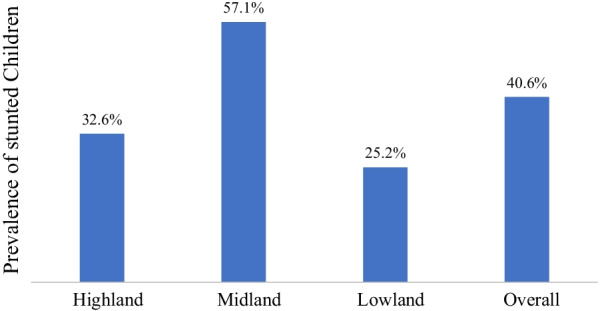
Prevalence of stunting among children across the three agro-ecological zones, Meta District, March 2017

Stunting is also more common among all agro-ecological settings namely high-land, mid-land, and low-land. However, stunting was more common among children from PSNP and NPNSP households living in the mid-land areas compared to their counterparts (high-land and low-land). The prevalence of stunting in the high-land, mid-land, and low-land was  32.6%, 57.1%, and 25.2% respectively (Fig. 4).

### Predictors of stunting among children aged 6–59 months

In multivariable binary logistic regression analysis, maternal factors such as lack of maternal education, women’s inability to make decision on households issues, lack of fourth antenatal care visit, practicing of hand washing during the critical periods, PSNP membership status, agro-ecological zone and child-related factors such as history of frequent childhood illness, lack of exclusive breastfeeding, eating less diversified foods, child’s age (being 24–59 months) and sex(being male) were identified as predictors of stunting.

Children who were born to mothers who have no formal education were 3.39 times [(AOR = 3.39; 95% CI (1.12, 5.11)] more likely to be stunted than children who were born to educated mothers. Stunting was 3.5 times [(AOR = 3.48; 95% CI (2.36, 5.12)] more common among children whose mothers could not make decision at household level on household matters than their counterparts. Children who were born to mothers who did not complete their fourth antenatal care visit (ANC IV) during their pregnancy were 4.2 time [(AOR = 4.2; 95% CI (2.5, 6.8)] more likely to be stunted compared with their counterparts. The odds of stunting was reduced by 54% [(AOR = 0.46; 95% CI (0.28, 0.76)] among children from households who were practicing hand washing during the critical periods (before eating and preparation of food, before child feeding, after use of latrine and after disposing of infant stool) compared with their counterparts. The odds of stunting were 1.94 times [(AOR = 1.94; 95% CI (1.12, 3.35)] more likely among children from mid-land ecologic zone compared with those form the high-land. In contrary, the odds of stunting were reduced by 73% [(AOR = 0.27; 95% CI (0.16, 0.45)] among children from low-land agro-ecologic zone compared with those from the high-land. The odds of stunting were nearly two times [(AOR = 1.82, 95% CI (1.14, 2.89)] among children whose parents are beneficiary of PSNP when compared with their counterparts.

Children who were repeatedly affected with childhood illness were 8.4 times [(AOR = 8.41; 95% CI (4.58, 12.76)] more likely to be stunted than their counter parts. The odds of stunting were nearly 4 times [(AOR = 3.6; 95% CI (2.3, 4.8)] higher among children who were not exclusively breastfed compared with their counterparts. Children who have consumed less diversified foods were nearly five times [(AOR = 4.7; 95% CI (3.0, 7.4)] more likely to be stunted compared to those who have fed on adequately diversified foods. Children in the age bracket of 24–59 months were three times [(AOR = 3.2; 95% CI (1.6, 6.3)] more likely to be stunted compared with younger ones. The odds of stunting were 1.73 times higher [(AOR = 1.73; 95% CI (1.18, 2.53)] among male children compared with their counterparts (Table 3).

**Table 3:** Predictors of stunting among children aged 6–59 months in Meta District, 2017

Variables (*n* = 1555)	Stunting		COR (95% CI)	AOR (95% CI)
	Yes (#, %)	No (#, %)		
*Maternal education*				
No formal education	594 (43.2)	780 (56.8)	2.71: (1.86, 3.94)**	3.39: (1.12, 5.11)**
Have formal education	38 (22)	135 (78)	1	1
*Hand washing practice*				
Yes	458 (36.3)	804 (63.7)	0.39: (0.3, 0.505)**	0.46: (0.28, 0.76)**
No	174 (59.4)	119 (40.6)	1	1
*Child’s illness*				
Yes	383 (70.3)	162 (7.7)	7.23: (5.73, 9.12)**	8.41: (4.58, 12.76)**
No	249 (24.7)	761 (75.3)	1	1
*Breastfeed in the first 6 months*				
No exclusively breastfed	510 (56.9)	386 (43.1)	5.82: (4.59, 7.37)**	3.6: (2.3, 4.8)**
Exclusively breastfed	122 (18.5)	537 (81.5)	1	1
*Woman’s participation in decision-making*				
No	503 (55.3)	406 (45.7)	4.97: (3.93, 6.27)**	3.48: (2.36, 5.12)**
Yes	129 (20)	517 (80)	1	1
*Antenatal care IV*				
No	465 (52.2)	426 (47.8)	3.25: (2.61, 4.04)**	4.2: (2.5, 6.8)**
Yes	167 (25.2)	497 (74.8)	1	1
*Child’s age*				
6–23 months	91 (17.3)	436 (82.7)	1	1
24–59 months	541 (52.6)	487 (47.4)	5.32: (4.12, 6.88)**	3.2: (1.6, 6.3)**
*Minimum dietary diversity*				
Less than 4 food groups	218 (31.9)	465 (68.1))	2.3: (1.83, 2.9)**	4.7: (3.0, 7.4)**
4 food groups and more	146 (16.7)	726 (83.3)	1	1
*Agro ecological zone*				
High-land	177 (32.6)	366 (67.4)	1	1
Mid-land	358 (57.1)	269 (42.9)	1.69: (1.52, 1.93)*	1.94: (1.12, 3.35)*
Low-land	97 (25.2)	288 (74.8)	0.25: (0.19, 0.34)**	0.27: (0.16, 0.45) **
*Father education*				
No formal Education	502 (43.4)	656 (56.6)	1.48: (1.17, 1.89)**	1.22: (0.77, 1.99)
Have formal Education	130 (33.9)	253 (66.1)	1	1
*Family size*				
≥ 5 family members	455 (44.3)	573 (55.7)	1.57: (1.26, 1.95)**	1.02: (0.66, 1.60)
< 5 Family members	177 (33.6)	350 (66.4)	1	1
*Sex of the child*				
Male	323 (42.6)	436 (57.4)	1.17: (0.95, 1.43)	1.73: (1.18, 2.53)**
Female	309 (38.8)	487 (61.2)	1	
*Membership*				
PSNP	373 (47.7)	409 (52.3)	1.81: (1.48, 2.23)**	1.82: (1.14, 2.89)*
NPSNP	259 (33.5)	514 (66.5)	1	1

*AOR* adjusted odds ratio, *CI* confidence interval, *COR* crude odds ratio

*p value < 0.05; **p value < 0.001

## Discussion

The nutritional status of children is affected by different interwoven factors which may vary from location to location, and inter-households. This study was aimed at assessing the prevalence of stunting and its predictors among children aged 6–59 months living in households enrolled in PSNP and those who were not-enrolled in the program in Meta District of east Harerge zone of eastern Ethiopia. Accordingly, the prevalence of stunting was 47.7% among children aged 6–59 months from PSNP beneficiaries and 35.5% for children who belong to NPSNP beneficiary households, respectively. Stunting was significantly associated with maternal factors such as lack of maternal education, women’s inability to make decision, agro-ecological zone, non-attendance of fourth antenatal care visit, practicing of hand washing during the critical periods, and being a beneficiary of PSNP and child-related factors such as child’s illness, lack of excusive breastfeeding, eating less diversified foods, child‘s age (being 24–59 months old) and sex.

In this study, the prevalence of stunting among children aged 6–59 months in PSNP beneficiary and NPSNP beneficiary households was 47.7% and 33.5%, respectively. This finding indicates a significant difference among the comparison groups. Similar finding was reported from Saesie Tsaeda Emba district, Tigray region, northern Ethiopia, where stunting is more common among food insecure households. The prevalence of stunting observed in Saesie Tsaeda Emba district of PSNP beneficiary and non-PSNP households was 52.1% and 46.1%, respectively [21]. Another national-based study reported that the prevalence of stunting was higher among PSNP users (47%) compared with NPSNP users (41.8%), respectively [3]. This difference might be due to the fact that PSNP beneficiary households are known to be at risk of food insecurity compared with their counterparts, NPSNP beneficiary, who are supposed to be relatively better-off. The cumulative effect of lack of food together with poor socio-economic attributes could aggravate the occurrence of chronic form of malnutrition among children from households benefiting from PSNP [22]. It is important that the existing PSNP needs to be upgraded to the level which can satisfy the balanced food needs of the poor segment of the population of interest.

Stunting is significantly associated with lack of maternal education. The odds of stunting were nearly 4 times higher among children who were born to mothers who have no formal education compared to their counter parts. Similar finding was reported from South Wollo community, northern part of Ethiopia [23], Fadis District community, Hararghe Zone eastern Ethiopia [22] and study from Saesie Tsaeda-Emba District community, Tigray of north Ethiopia [21], and from Tanzania [24]. This might be attributed to the fact that child caring is mainly responsibility of the mother/care giver in Ethiopian context which is added to lack of knowledge of implementing appropriate to age child feeing practices and understanding of the adverse effects of undernutrition on children in the later life.

Children who were born to mothers who were not able to make decision on the household matters were nearly 5 times more likely to have an increased odd of stunting compared with children who were born to empowered women who can make decision on their own. This finding was consistent with Maharashtra’s study of Indians [6]. This might imply that when women are empowered to decide on household’s matters the likelihood of caring for their children increases since they have access to household resources enhancing food accessibility for the household members.

Overall, the prevalence of stunting in the high-land, mid-land, and low-land were 32.6%, 57.1%, and 25.2%, respectively. The odds of stunting were 1.94 times higher among children from mid-land agro-ecologic zone compared with those form the high-land. In contrary, the odds of stunting were reduced by 73% among children from low-land agro-ecologic zone compared with those from the high-land. The variation in the prevalence of stunting could be the effect of climatic zones on rain fall which might affect the crop production and thereby subject the community to lack of adequate food. For instance, children living in mid-land are at risk of stunting probably due to less fertility of the mid-land itself giving rise to inadequate food production. The reduction in the prevalence of stunting by more than half among children from low-land agro-ecologic zone is controversial and requires further larger scale study although population living in low-land geographic area are at risk of undernutrition including stunting resulting from low rainfall and infectious diseases like malaria [25]. However, precaution needs to be taken upon interpretation of these results and further research should be conducted using appropriate analytical study design to reach at conclusion.

Lack of proper antenatal care follow-up is also found to be associated with stunting. The odds of stunting were 7 times higher among children being delivered from mothers who did not attend their fourth ANC follow-up compared with their counterparts. Similar finding was reported from Yemen [7]. This might be due to the fact that child nutrition-related issues are often addressed during pregnancy when pregnant women visit health facilities more frequently and get information as how to feed their children after delivery to prevent the occurrence of child undernutrition.

The odds of stunting were reduced by 62 %, among children from households who practiced hand washing during the critical periods. This finding was consistent with study finding from northern part of the country [21]. This might be due to the efficiency of hand washing practice which can reduce pathogens that cause disease and thereby reduce stunting. It also implies in addition to other factors related chronic malnutrition an attempt needs to be made in scaling-up such cost effective intervention in reducing the magnitude of chronic malnutrition.

The odds of stunting were nearly two times among children from households who are benefiting from PSNP compared to their counterparts. It is obvious that beneficiaries of the PSNP are enrolled into the program based on their low economic background among other criteria to survive the effects poverty through implementation of PSNP by the support given to them by the national and local governing bodies. However, this result needs to be carefully interpreted since the effect of PSNP may take time to enable the beneficiaries able to absorb shocks and thereby improve their nutritional status. It also requires vigorous research design to see the effect of PSNP on nutritional status of the program beneficiaries. Yet we did not have strong evidence on the relationship between nutritional status of children and PSNP though we have a few evidence that PSNP membership had a significant effect on household dietary diversity and minimal effect on women’s body mass index and children’s nutritional indices including stunting [3, 26].

Children who had history of illness in the last 2 weeks prior to data collection period were nearly 9 times more likely to be stunted than their counterparts. This finding was comparable with community-based study done in, northwest Ethiopia [4] and Latin America Amazon, Western Brazilian [27]. This might be due to increase in body’s calorie requirement during illness and destruction of tissue as the result of the micro-organisms among stunted children.

Stunting was nearly 6 times more common among children of mothers who have not exclusively breastfed their children in the previous six months compared with their counter parts. This finding is agreement with community-based study from Somali region of eastern Ethiopia [28], Saesie Tsaeda Emba district of north Ethiopia [21] and in rural Cambodia [29]. This might be explained in such a way that breast milk has substantial amount of anti-infective substances that can safeguard children from the occurrence of infection thereby break malnutrition—infection complex cycle.

In this study, there is also a significant association between minimal dietary diversity and stunting. The odds of stunting were nearly 7 times higher among children who have consumed less diversified foods compared with their counterparts. This result was comparable with findings of studies done in Saesie Tsaeda Emba district of northern Ethiopia [21], Somali region of eastern Ethiopia [28] and Cambodia [29]. This result implies that diversified foods are very important for child growth and development during the age of 6–59 months to provide all micronutrients and macronutrients for the body requirements.

The risk of stunting increases as the child’s age increases. In this study, children aged 24–59 months were 6. 9 times more likely to be stunted compared to children aged 6–23 months. This result was in line with the national figure [3] and study done in Belesa District [4], where children aged 24–35 months were more likely to be stunted when compared to earlier age group. This might be due to lack of continued child feeding practice with diversified diet and may be related to poor breastfeeding practice before the age of 23 months.

Moreover, the odds of stunting were 1.73 times higher among male children compared with their counterparts. This finding was congruent with previous studies done in Ethiopia and Rwanda [8, 30]. The explanation might be due to social beliefs that females are an asset to the family and given more value than males in the studied community.

In this study, the observed prevalence of stunting for both population categories is alarming indicating its high or serious level as per the WHO recommendation [31] necessitating appropriate interventions to mitigate its short- and long-term consequences. All modifiable factors identified as having an association with stunting need to be given due attention by nutrition program planners and stakeholders through designing all relevant nutrition interventions.

This study could have the following limitations. Firstly, mothers/caregivers might have wrongly reported their children’s age which could have resulted in inappropriate estimation of the outcome measure of interest, stunting . However, age was estimated carefully based on mother’s/caregiver’s unforgettable events such as public and religious holidays of the year using local calendar for those who did not have birth certificates and health service-related evidence such as immunization card. Secondly, recall bias could be introduced upon collecting data on some past background variables such as 24 hr recall questions in which case an interactive 24 hr recall method was used to minimize such a bias. Thirdly, an anthropometric measurement error could potentially affect the results of this study due to both random and systematic error. Nevertheless, all important efforts such as maintenance of five contact points while measuring length/height of the child, extensive training of data collectors including standardization procedure before deploying data enumerators to the field and refreshment training for data enumerators sometimes after starting data collection was given to minimize the errors.

## Conclusions

The prevalence of stunting is high among children from beneficiaries of PSNP compared with non-beneficiaries in the study area. Stunting was statistically significantly associated with maternal education, decision-making power, hand washing practices during critical periods, agro-ecologic set-up, antenatal care service and PSNP membership status, exclusive breastfeeding practice, minimum dietary diversity, child’s illness, sex and age. Thus, intensifying women’s decision-making on household’s matters including health services and improving infant and young child’s feeding practices should be given attention to reduce the impacts of stunting on child's health by all relevant actors working in the area of child health program. Furthermore, promotion and scale-up should be made to intensify the impact of PSNP on nutritional status of children born to PSNP beneficiary households. The authors also recommend doing similar research using prospective longitudinal study design to follow-up the children in an attempt to determine the prevalence of stunting to overcome the problem of child’s age misclassification in rural settings.

## Data Availability

The datasets used and/or analyzed in this study will be made available by the corresponding author upon reasonable request.

## Abbreviations

AOR: Adjusted Odds Ratio
BCC: Behavioral Change Communication
COR: Crude odds ratio
EDHS: Ethiopia Demographic Health Survey
HAB: Household Asset Building Program
HAZ: Height-for-age *Z*-score
HH's: Households
HP: Health post
IYCF: Infant and Young Child Feeding
NNP: National Nutrition Program
NPSNP: Non-Productive Safety Net Program
PSNP: Productive Safety Net Program
UNICEF: United Nations Children’s Emergency Fund
WAZ: Weight-for-age *Z*-score
WHO: World Health Organization
WHZ: Weight-for-height *Z*-score
